# Glass-(nAg, nCu) Biocide Coatings on Ceramic Oxide Substrates

**DOI:** 10.1371/journal.pone.0033135

**Published:** 2012-03-12

**Authors:** Leticia Esteban-Tejeda, Francisco Malpartida, Luis Antonio Díaz, Ramón Torrecillas, Fernando Rojo, José Serafín Moya

**Affiliations:** 1 Department of Biomaterials and Bioinspired Materials, Materials Science Institute of Madrid (ICMM-CSIC), Cantoblanco, Madrid, Spain; 2 Department of Microbial Biotechnology, National Center for Biotechnology (CNB-CSIC), Cantoblanco, Madrid, Spain; 3 Nanomaterials and Nanotechnology Research Center (Centro de Investigación de Nanomateriales y Nanotecnología-Spanish-National-Research-Council-Universidad de Oviedo-Principado de Asturias, (CINN-CSIC-UNIOVI-PA), Parque Tecnológico de Asturias, Llanera, Spain; KU Leuven, Belgium

## Abstract

The present work was focused on obtaining biocide coatings constituted by a glassy soda-lime matrix containing silver or copper nanoparticles on ceramic (alumina and zirconia based) substrates. Both glassy coatings showed a high biocide activity against Gram−, Gram+ bacteria and yeast, reducing cell numbers more than three logarithms. Silver nanoparticles had a significantly higher biocide activity than copper nanoparticles, since the lixiviation levels required to reduce cell numbers more than 3 logarithms was of almost 1–2 µg/cm^2^ in the case of silver nanoparticles, and 10–15 µg/cm^2^ for the copper nanoparticles.

## Introduction

The problem of biofouling or biocontamination is a great concern in a wide range of applications such as surgical equipment and protective apparels in hospitals, medical implants, water purification systems, industrial and marine equipment, oil rigs biosensors, food packaging, food storages or textiles. In this sense, there is a need of effective and non toxic antifouling coatings to prevent the settlement and growth of microorganisms, which are associated to infections, serious complications in hospitals, food industries and community settings [1], [2]. For many years, organic compounds of tin, specially tributyltin (TBT), were widely used. However, its severe toxicity, even in a few nanograms per liter, led to regulate its use internationally since 1990. Nowadays, there is a great interest in developing new non toxic inorganic biocide coatings [3]. In the last decade several investigations have dealt with coating mainly based upon silicones and fluopolymers. However, these coatings have drawbacks: high price, mechanical fragility, difficult application and persistence [3]. Currently, more than 18 chemicals are used as antifouling agents throughout the world but only nine of them are approved for use by Health and Safety Executive (HSE) in the United Kingdom [4]–[7].

The antimicrobial properties of silver ions were known since ancient times and they are widely used as bactericides in catheters, burn wounds or dental work [8]. Researchers have also recommended the use of silver and copper ions as superior disinfectants for wastewater generated from hospitals containing infectious microorganisms [9], [10]. The emergence of nanoscience and nanotechnology in the last decade presents opportunities for exploring the bactericidal effect of nanostructured materials containing silver and copper nanoparticles. The bactericidal effect of metal nanoparticles is not merely due to the release of metal ions in solution, and it has been attributed to their small size and high surface to volume ratio, which allows them to interact closely with microbial membranes [11], [12]. The antimicrobial properties of silver nanoparticles are well-established and several mechanisms for their bactericidal effects have been proposed [13]–[16]. On the contrary, only a few studies have reported the antibacterial properties of copper nanoparticles, showing that copper nanoparticles have a significant promise as bactericidal agent [17], [18]. In addition, there is a great controversy in the literature about the biocide efficiency of silver and copper nanoparticles. Yoon et al. [19] reported the antibacterial effect of silver and copper nanoparticles using single representative strains of *Escherichia coli* and *Bacillus subtilis* concluding that *E. coli* is more resistive to nanoparticles than *B. subtilis* is. Ruparelia et al. [18] reported that *E. coli* depicts higher sensitivity to the silver nanoparticles than to the copper nanoparticles. On the contrary, the Gram+ *B. subtilis* was more sensitive to the copper nanoparticles than to the silver nanoparticles and it has also been found that all the *S. aureus* strains exhibited identical sensitivity to silver and copper nanoparticles so no strain specificity was observed. Hence, the bactericidal efficiency of nanoparticles is not solely dependent on the structure of the bacterial membrane.

Nanoparticles with biocide activity containing silver or copper can be immobilized and coated onto surfaces in order to obtain a biocide coating. Some research has been focused on this field, i.e. the development of cement-based biocide coating containing copper nanoparticles for controlling algal growth in water distribution canals [20], biocide coatings with Ag/SiO_2_ core shell nanoparticles [21] or the synthesis of potential antifoulings functionalized with copper nanoparticles [22]. However, the incorporation of silver or copper nanoparticles into a glassy matrix is still poorly understood, although it has several advantages such as a controlled lixiviation of the nanoparticles, which increases the durability of the biocide coating and reduces its toxicity and the health problems derived of nanoparticles manipulation [23], [24]. In this work we have selected silver to obtain biocide coatings in medical implants and food packaging or storage. Copper, which presents toxicity similar to that of zinc [25], [26], was selected for applications in which the cost is important.

Alumina and zirconia were chosen as ceramic substrates due to the fact that they are widely used in medical implants (dental implants, knee or hip replacements) [27] as well as in pharmacy and food industries [28]–[31]. We have also designed a novel method to determine the fraction of nanoparticles lixiviated from the coating in order to compare the biocide efficiency of the coating. In addition, the route of synthesis for these biocide and non toxic coatings is simple, reliable and easy to scale up for industrial production at low cost. The biocide activity of the coatings was studied against *E. coli* (Gram-negative bacteria), *M. luteus* (Gram-positive bacteria) and *I. orientalis* (yeast). The mechanical stability of the coatings is also reported.

## Materials and Methods

### Materials

The starting materials were as follows: i) a commercial soda-lime glass from the SiO_2_-Na_2_O-K_2_O-CaO-MgO-B_2_O_3_ system with the following chemical composition (wt%): 70.2 SiO_2_, 15.8 Na_2_O, 7.1 CaO, 3.2 MgO 1.06, B_2_O_3_ and 0.05 K_2_O with a deformation point ∼668°C, and ii) a sepiolite-nCu supplied by TOLSA S.A. with a content of copper of 26wt.%. This sepiolite-nCu was fully characterized by X-Ray diffraction (XRD), ultraviolet-visible absorption spectroscopy (UV-VIS spectroscopy) and transmission electron microscopy (TEM) in a previous work [24] and iii) vitellinate/nAg (Batch n° 127, ARGENOL S.L.), which is a protein of high molecular weight with a particle size distribution of d_50_≈10±2 nm. This sample was fully characterized in a previous work [32] by differential thermal analysis (DTA), thermogravimetry (TG), X-Ray diffraction (XRD), ultraviolet-visible absorption spectroscopy (UV-VIS spectroscopy) and transmission electron microscopy (TEM). The chemical analysis was determined by inductively coupled plasma (ICP) and was found to be 20 wt.% of silver and 7.6 wt.% of sodium oxide. The rest are organic compounds (C_n_-H_m_-N_x_).

The alumina (99.9% purity, 99% theoretical density, biomedical grade) and the tetragonal zirconia-based composite (Ce-TZP/nAl_2_O_3_) with the following composition (wt%): 65.20% ZrO_2_, 8.55% CeO_2_, and 24.88% Al_2_O_3_; 99% theoretical density, (biomedical grade) ceramic substrates were supplied by Nanoker S. L.

### Coating

Biocide soda-lime glass powders containing 20 wt% Cu^0^ or Ag^0^ were obtained as it is hereby described: glass (<32 µm) and the corresponding fraction of sepiolite-nCu or vitellinate-nAg were homogeneously blended in isopropyl alcohol overnight under constant stirring of 30 rpm. After that, the suspensions were dried at 60°C for 4 h, the homogeneous mixtures were uniaxially pressed into pellets (Ø∼10 mm) at 250 MPa.

Then, they were sintered in air in two-steps, by heating at a rate of 3°C/min first to 500°C, and then to 725°C, and holding for 1 h in the case of vitellinate-nAg. In the case of sepiolite-nCu, there is no need to burn organic compounds so the heating rate was 10 C/min to 725°C and the hold was 1 h in argon atmosphere to avoid the copper oxidation. Tubular electrical furnace and zirconia crucibles were used. The obtained glass-n(Cu, Ag) pellets were milled down to <32 µm in an agata planetary ball mill. These powders were fully characterized by XRD using a Bruker D8 diffractometer using CuKα radiation working at 40 kV and 30 mA in a step-scanning mode from 5 to 70° with a step width of 0.0288 and a step time of 2.5 s, by scanning electron microscopy (SEM) (Hitachi S-4300) and transmission electron microscopy (TEM) (JEOL FXII at 200 kV). Optical absorption spectrum was measured in a range from 200 nm to 800 nm, using a JASCO UV-Vis V-660 spectrophotometer to determine the surface plasmon resonance of silver or copper nanoparticles. The hot stage measurements were carried out in a high temperature HESSE microscope at 5°C/min on alumina support. The chemical analyses of coatings were performed in a Perkin Elmer spectrometer model 2100. Finally, the thermal expansion coefficients were measured in a BÄHR THERMOANALYSE model DIL 802 in air atmosphere for the silver containing glass samples and in argon atmosphere for the copper ones.

Coatings on alumina and zirconia plates with 10×25×1 mm dimensions were done by deposition of these powders (0.2 g) from homogeneous suspension in acetone (20 mL) and subsequently air-dried at 40°C. Afterwards, the coated plates were heated at 10°C/min in the corresponding air or argon atmosphere at different temperatures ranging from 740 to 1150°C for 1 h, depending on the hot stage optical microscopy observations.

### Characterization of the coating

Surface and cross-section morphology and composition of the coated samples were analyzed by scanning electron microscopy (SEM) coupled with energy dispersive spectroscopy (EDS) (Hitachi S-4300). XRD analyses of samples were carried out in a Bruker D8 diffractometer using CuKα radiation working at 40 kV and 30 mA in a step-scanning mode from 10° to 70° with a step width of 0.028° and a step time of 2.5 s.

### Biocide activity test

**Figure 1:**
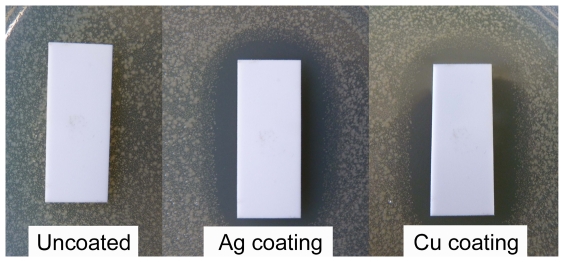
Diffusion method for *E. coli* for the uncoated and coated alumina substrates obtained at 740°C for n-Ag glass and at 850°C for the n-Cu glass. A similar result was obtained for zirconia substrates.

The antimicrobial benefits of the coatings were evaluated against three different microorganisms: *Escherichia coli JM110* (Gram-negative bacteria), *Micrococcus luteus* (Gram-positive bacteria) and *Issatchenkia orientalis* (yeast). The microorganisms were incubated overnight at 37°C in a suitable liquid media [i.e., Luria Bertani (LB) for *E. coli* and *M. luteus*, or yeast extract dextrose (YEPD) for *I. Orientalis*]. One µL from each overnight culture were diluted into 1 mL of LB or YEPD and 50 µL of the diluted cultures were added on 4 mL of melted Soft Nutrient Agar (SNA, containing LB or YEPD and 0.6% agar) and poured onto Petri dishes containing LB-agar or YEPD-agar (1.5% agar). Subsequently, the different coated plates were deposited over the solidified SNA agar containing the microorganisms. A biocide free coating (culture containing the corresponding nutrient, microorganisms and uncoated plate) was used as control. The cells surviving below the coated or uncoated plates were measured after 24 hours. To this purpose, a glass capillary (Ø∼1 mm) was used to remove a small area of SNA, containing the microorganisms, below the glass-coated and the corresponding uncoated ceramic substrates (figure 1). This test was performed in triplicate. The pieces of SNA gel containing the microorganisms were deposited on 500 µL of a buffer solution of PBS (Phosphate Buffered Saline) and ultrasonicated during 2 min to release the microorganisms from the SNA. After decanting the solutions for 10 minutes in ice (to avoid the microorganism growth), 100 µL were taken to perform serial dilutions. The number of cells in each dilution was counted by homogeneously spreading 100 µL of each dilution in LB or YEPD agar plates and incubating the plates at 30°C for 24 hours. The colonies formed by the microorganisms on the plates were counted. In parallel, the aqueous fraction of the SNA which contains the silver or the copper released nanoparticles at the end of the growing period was obtained by cutting the areas of SNA which were in contact with the coated ceramic substrates, and extracting the aqueous fraction by centrifugation in eppendorf tubes containing glassy wood at the bottom. The content of silver or copper was determined by inductively coupled plasma (ICP) (ICP Perkin Elmer mod. optima 2100 DV).

## Results

### Coating Characterization

The chemical analyses of glass-nAg (20wt%) and glass-nCu (20wt%) are showed in table 1.

**Table 1:** Chemical composition of glass-nAg (20wt%) and glass-nCu (20wt%) coatings.

	SiO_2_	CaO	Na_2_O	MgO	K_2_O	Al_2_O_3_	B_2_O_3_	Fe_2_O_3_	Cu	Ag
n-Ag coating	46.50	4.60	18.1	1.83	0.03	1.02	1.00	0.17	-	20.05
n-Cu coating	52.99	1.66	5.64	14.58	2.40	1.27	0.23	0.43	20.08	-

The thermal expansion coefficients corresponding to: glass-nAg (20wt%), glass-nCu (20wt%) and to both ceramic substrates are given in table 2.

**Table 2:** Thermal expansion coefficients for the n-Ag glass, n-Cu glass, Ce-TZP/nAl_2_O_3_ and alumina.

	Thermal expansion coefficient: α_10-800_ (°C^−1^)
glass-nAg	11·10^−6^
glass-nCu	9.9·10^−6^
α-Al_2_O_3_	7.5·10^−6^
Ce-TZP/nAl_2_O_3_	12·10^−6^

**Figure 2:**
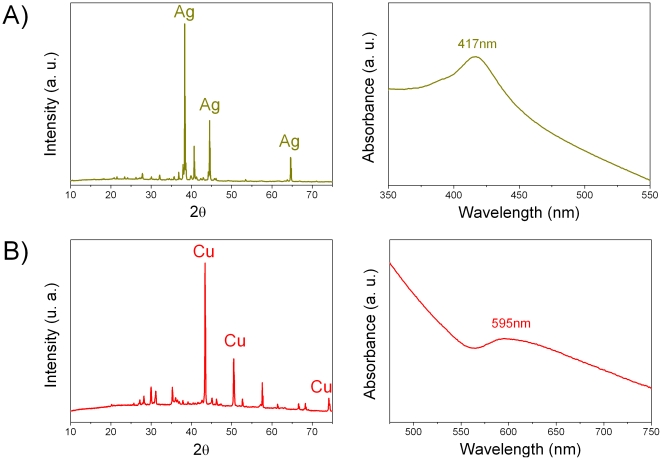
XRD patterns and UV-vis spectra corresponding to: A) n-Ag glass coating obtained at 740°C and B) n-Cu coating obtained at 850°C.

The XRD patterns and the UV-vis spectra of the obtained coatings are represented in figure 2. We can observe the Bragg's reflections (2θ = 38.2, 44.4 and 64.6°) of metallic silver (JCPDS 87-0720) for the glass-nAg coating (figure 2 A) and the diffraction maxima (2θ = 43.39°, 50.50° and 74.19°) corresponding to cubic copper metal crystals (JCPDS 85-1326) for the glass-nCu coating (figure 2 B).

**Figure 3:**
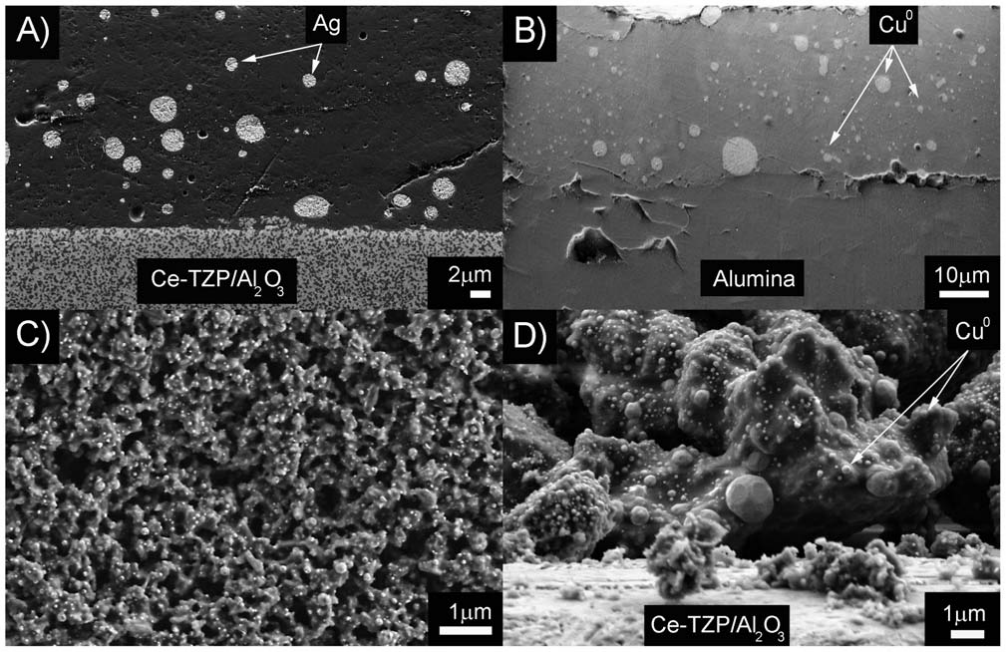
Scanning electron micrographs corresponding to: A) cross-section of n-Ag glass coating on Ce-TZP/nAl_2_O_3_ substrate sintered at 740°C, 1 h in air atmosphere; B) cross-section of n-Cu glass coating on alumina substrate sintered at 1150°C, 1 h in argon atmosphere; C) top view and D) cross-section of n-Cu glass coating on Ce-TZP/nAl_2_O_3_ substrate sintered at 850°C, 1 h in argon atmosphere.

**Figure 4:**
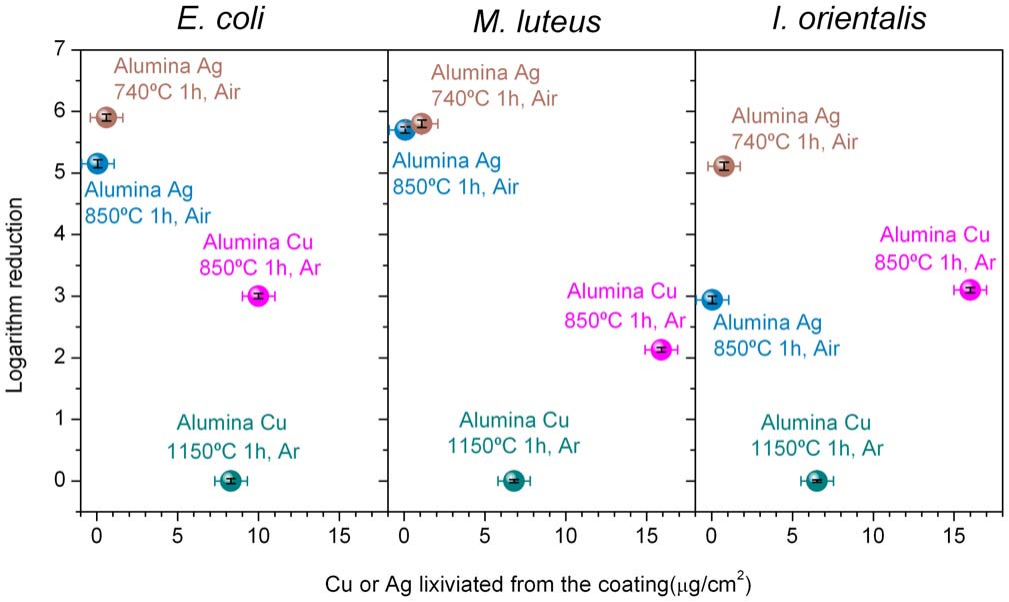
Logarithm of reduction of *E. coli*, *M. luteus* and *I. orientalis* for the coatings versus the total of silver or copper lixiviated per area unit.

The microstructures of the different coatings are shown in figure 3. The strong biocide effect of both glass-nAg and glass-nCu coatings can be clearly observed using the diffusion method for *E. coli* showed in figure 1. Figure 4 shows the logarithm of reduction for *E. coli*, *M. luteus* and *I. orientalis* plotted versus the total µg of silver or copper per area unit (cm^2^). The logarithm of reduction was calculated as follows:
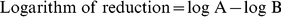
(1)where A is the average number of viable cells (CFU) from inoculum control after 24 h, and B is the average number of viable cells (CFU) from the broth containing the biocide agent after 24 h.

## Discussion

In the diffraction patterns of copper coating no peaks corresponding to copper oxide appeared, indicating that no oxidation of metallic copper nanoparticles takes place. This is consistent with the corresponding UV-vis spectra (figure 2), where the surface plasmon resonance corresponding to silver (417 nm) and copper (595 nm) metallic nanoparticles are observed. A partial crystallization of the glass took place due to the thermal treatment, as indicated by the appearance of peaks corresponding to several calcium sodium silicate compounds: NaCa_2_Si_3_O_8_OH, (JCPDS 12-0238), Na_2_Ca_2_Si_2_O_7_•H_2_O (JCPDS 21-1349), Na_2_Ca_3_Si_6_O_16_ (JCPDS 77-0386) and Na_2_Si_3_O_7_ (JCPDS 86-0436).

As it can be observed in figure 3 A a perfect almost free porous glass coating is obtained in the case of glass-nAg coatings on alumina and zirconia based substrates heated in air atmosphere at 740°C. The only observed difference was the appearance of some cracking in the case of glass-nAg coating on the alumina substrate. This is due to the large gap in the thermal expansion coefficients (Δα = 3.5°C^−1^).

The silver nanoparticles are homogeneously distributed in the glassy matrix with a particle size ranging from 20 to 90 nm. Some agglomerates (0.5–2 µm) also are present (figure 3 A). In the case of the glass-nCu coating, due to the decreased content in modifiers oxides and the higher content in silica, compared with the glass-nAg coating, (61.62 vs 46.5wt%) in the glassy phase, to obtain a free porous coating the required temperature was found to be 1150°C (figure 3 B) which was determined by the hot stage study. At lower temperature (850°C), the coating shows open porosity (∼40%) as it can be seen in figures 3 C and 3 D. This porosity facilitates the required lixiviation of copper during the biocide test.

Considering the nowadays open controversy about the biocide efficiency of silver and copper nanoparticles [18], [19], it is important to underline that our experimental results clearly point out that the silver nanoparticles are significantly more active than the copper nanoparticles. In the case of *E. coli*, the lixiviation of ∼1 µg/cm^2^ of silver led to a strong biocide activity that reduced cell numbers by almost 6 logarithms. Conversely, to reach a logarithm reduction of ∼3, the lixiviation of 10 µg/cm^2^ of copper nanoparticles was required. Similar results were obtained for *M. luteus* and *I. orientalis*. From these results it can be stated that dense coating has biocide activity only for silver nanoparticles but not for copper nanoparticles. The lixiviation of copper in alumina glass-nCu coating (figure 4) was of ∼7 µg/cm^2^, which is not enough to have biocide activity. To be an active biocide coating an open porosity of ∼40% is required (figures 3 C, 3 D and 4. In all the studied samples the levels of Ag and Cu are below the toxic limit: 30 ppm is the cytotoxic limit established by Panacek et al. [33] in the case of silver nanoparticles for human fibroblast and according to Chen et al. [25], 413 ppm for the case of copper nanoparticles.

In summary, following a simple sedimentation route a glass-nAg (20 wt%) biocide coating has been obtained at 740°C, 1 h in air on alumina and zirconia based substrates. Similarly, a glass-nCu (20 wt%) biocide coating with an open porosity of ∼40% has been obtained at 850°C, 1 h in argon on alumina and zirconia based substrates. Both glass coatings have a high biocide activity versus Gram−, Gram+ bacteria and yeast. The reported results also show that silver nanoparticles have a significantly higher biocide activity than copper nanoparticles, since to reach a bactericidal effect that reduced cell numbers by >3 logarithms, the required lixiviation in the case of silver nanoparticles was of almost 1–2 µg/cm^2^, while for copper nanoparticles the needed lixiviation was almost ten times higher (10–15 µg/cm^2^).
